# Zollinger-Ellison syndrome associated with neurofibromatosis type 1: a case report

**DOI:** 10.1186/1471-2407-5-85

**Published:** 2005-07-21

**Authors:** Wan-Sik Lee, Yang-Seok Koh, Jung-Chul Kim, Chang-Hwan Park, Young-Eun Joo, Hyun-Soo Kim, Chol-Kyoon Cho, Sung-Kyu Choi, Jong-Sun Rew, Sei-Jong Kim

**Affiliations:** 1 Division of Gastroenterology, Department of Internal Medicine, Chonnam University Medical School, Hak-dong 8, Donggu, Gwangju, Korea; 2 Department of Surgery, Chonnam University Medical School. Hak-dong 8, Donggu, Gwangju, Korea

## Abstract

**Background:**

Neurofibromatosis type 1 is an autosomal dominant neurocutaneous disorder with characteristic features of skin and central nervous system involvement. Gastrointestinal involvement is rare, but the risk of malignancy development is considerable. Zollinger-Ellison syndrome is caused by gastrin-secreting tumors called gastrinomas. Correct diagnosis is often difficult, and curative treatment can only be achieved surgically.

**Case presentation:**

A 41-year-old female affected by neurofibromatosis type 1 presented with a history of recurrent epigastric soreness, diarrhea, and relapsing chronic duodenal ulcer. Her serum fasting gastrin level was over 1000 pg/mL. An abdominal CT scan revealed a 3 × 2-cm, well-enhanced mass adjacent to the duodenal loop. She was not associated with multiple endocrine neoplasia type 1. Operative resection was performed and gastrinoma was diagnosed by immunohistochemical staining. The serum gastrin level decreased to 99.1 pg/mL after surgery, and symptoms and endoscopic findings completely resolved without recurrences.

**Conclusion:**

Gastrinoma is difficult to detect even in the general population, and hence symptoms such as recurrent idiopathic peptic ulcer and diarrhea in neurofibromatosis type 1 patients should be accounted for as possibly contributing to Zollinger-Ellison syndrome.

## Background

Neurofibromatosis type 1 (NF1) is an autosomal dominant neurocutaneous disorder with an estimated prevalence of 2 to 3 cases per 10,000 population [1]. NF1 is characterized by neurocutaneous signs such as café-au-lait spots, axillary freckling, and cutaneous neurofibromas. Cognitive deficits and academic learning difficulties are the most common neurological complications of NF1 in childhood. Moreover, patients with NF1 are at an increased risk of developing nervous system neoplasms. Malignancy other than of nervous system origin can develop, with the prevalence of such malignant tumors reportedly being four times higher in NF1 patients than in the general population [2]. Gastrointestinal malignancy was reported to be associated with NF1 patients, with neuroendocrine tumors being the most prevalent [3]. However, there have been only one report about gastrinoma associated with NF1.

Zollinger-Ellison syndrome (ZES) is characterized by hypersecretion of gastrin from a gastrinoma that leads to gastric acid hypersecretion and, most notably, clinical symptoms of refractory peptic ulcer disease. Zollinger and Ellison were the first to report recurrent peptic ulcers of the jejunum associated with nonbeta islet cell tumors of the pancreas [4]. The diagnosis is based on a combination of criteria, including clinical presentation, serum gastrin level, acid-secretion test, and diagnostic imaging studies. Improvements in medical management using proton-pump inhibitors mean that gastric acid hypersecretion can be effectively controlled in most cases. Determining the presence of metastasis to regional lymph nodes or the liver to monitor malignant progression is important before definitive treatment. ZES in the form of multiple endocrine neoplasia type 1 (MEN1) needs special consideration, since the disease progression, presentation, and treatment differ from those in the sporadic form [5].

In this report, we describe a patient with NF1 who was diagnosed as ZES and successfully treated by curative surgery.

## Case presentation

A 41-year-old female NF1 patient was admitted for the evaluation of a 3-year history of recurrent epigastric soreness, heartburn, and diarrhea. Repeated endoscopic examinations revealed recurrent duodenal ulcers. The symptoms were relieved by proton-pump inhibitors, but recurred when the medication was withdrawn. She was previously diagnosed with NF1 based on clinical features and family history. Her first-degree relatives (i.e., brother, sister, and second son) were also affected by fully developed features of NF1. She had no cognitive dysfunction or learning disabilities, and showed normal intellectual development. A physical examination revealed several café-au-lait spots and multiple small nodules on the anterior chest and areolar area, and also multiple axillary freckles. A skin-nodule biopsy demonstrated characteristic neurofibromas.

**Figure 1:**
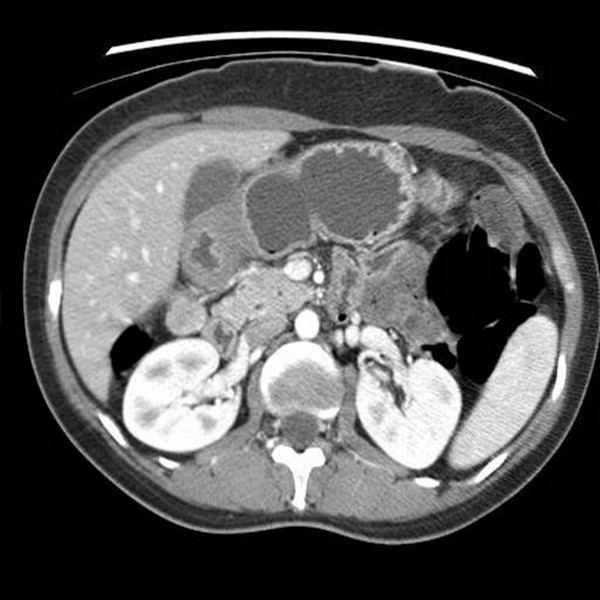
Abdominal CT scan showing a 3 × 2 cm, well-defined, uniformly enhanced mass adjacent to the duodenal loop.

On admission, upper gastrointestinal endoscopy revealed multiple shallow ulcers in the descending duodenum. The rapid urease test and urea breath test for *Helicobacter pylori *were both negative. The serum fasting gastrin level was >1,000 pg/mL and 837 pg/mL in two consecutive measurements. A secretin stimulation test was not performed due to the unavailability of secretin. As a diagnosis of gastrinoma was strongly suggested, radiologic evaluations were performed to locate the lesion. Abdominal computed tomography (CT) and magnetic resonance imaging (MRI) scans showed a 3 × 2-cm, clearly defined, well-enhanced mass adjacent to the duodenal loop in the subhepatic space (Figure 1). No metastatic lesions were observed in the liver or regional lymph nodes.

**Figure 2:**
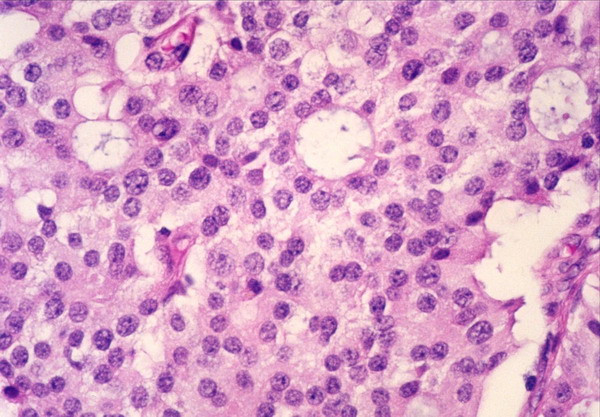
The pathologic findings were of a well-encapsulated solid mass containing cells with a diffuse, trabecular growth pattern. (×40, H&E).

**Figure 3:**
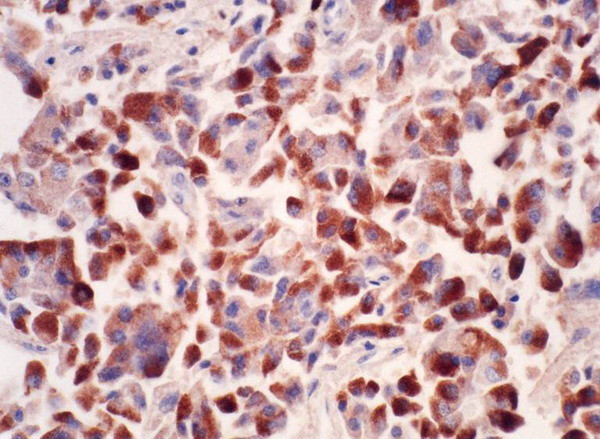
The tumor cells were strongly immunopositive for gastrin. (×250, avidin-biotin-peroxidase).

**Figure 4:**
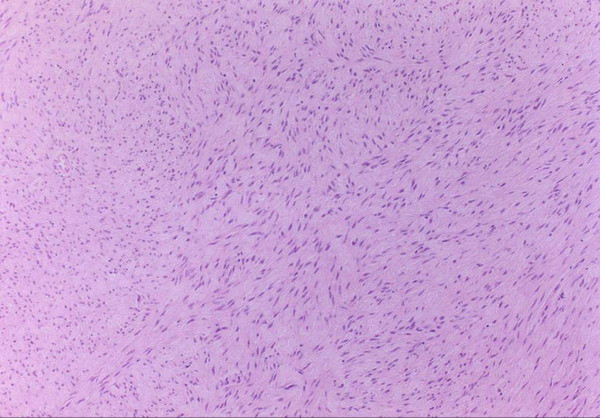
Biopsy of the multiple small nodules revealed the haphazard arrangement of uniform spindle cells in a collagenous stroma. (×100, H &E).

**Figure 5:**
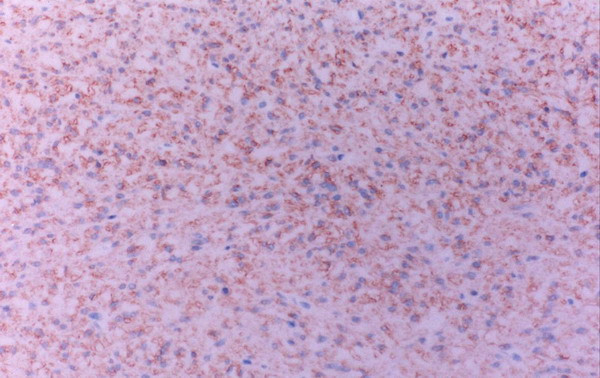
The tumor cells were immunopositive for C-kit (CD117). (× 100, H &E).

The association with MEN1 status was evaluated by hormonal and radiologic investigations of the parathyroid, pituitary, pancreas, and adrenal gland, which proved to be negative. As a definitive treatment, an intraabdominal mass of about 2.5 × 2 cm with a thick fibrous capsular outer layer was surgically isolated and completely resected from the right lateral border of the descending duodenum. Gastrinoma was finally diagnosed by immunohistochemical staining (Figures 2, 3). An incidental finding during the operation was numerous, small (<1.0 cm), whitish nodular masses on the serosal surface of the small bowel, many of which were enucleated. The pathologic diagnosis was gastrointestinal stromal tumor (GIST) with a low mitotic index as evidenced by C-kit positivity (Figures 4, 5). The serum gastrin level decreased markedly to 99.1 pg/mL at 1 month after the operation, and to 53.2 pg/mL 3 months later. There were no symptomatic or radiologic recurrences during a 14-month follow up.

## Discussion

NF1 is an autosomal dominant single-gene disorder with an estimated prevalence of 2 to 3 cases per 10,000 population [1]. NF1 is characterized by neurocutaneous signs, such as café-au-lait spots, axillary freckling, and cutaneous neurofibromatosis, and iris hamartomas [6]. Neurofibromas are benign peripheral nerve tumors composed of proliferating Schwann cells and fibroblasts. They present as multiple palpable, rubbery cutaneous tumors, and are generally asymptomatic.

In our case, the diagnosis of NF1 was established based on characteristic clinical features including dermatologic findings, pathological diagnosis of neurofibroma, and familial history of NF1 in first-degree relatives. These fulfilled the diagnostic criteria of NF1, and so further genetic testing was considered unnecessary [6]. There was a possibility that the skin lesions were associated with the MEN1 and that the gastrinoma occurred in the context of MEN1. However, meticulous laboratory and radiologic investigations indicated that the patient was not affected by MEN1.

NF1 patients have mutations of the NF1 gene on chromosome 17 [7]. The NF1 gene is a tumor-suppressor gene encoding a protein, neurofibromin, which functions in normal cells as a suppressor of the ras signaling cascades [8].

The development of malignancy in NF1 is frequently encountered, and can influence the outcome [9]. The prevalence of malignant tumors is reportedly four times higher in NF1 patients than in the general population [2]. Patients with NF1 are at an increased risk of developing central nervous system tumors, including optic pathway gliomas and brainstem and cerebellar tumors [10,11].

Gastrointestinal involvement in NF1 occurs at diverse locations, and its clinical features encompass variable manifestations including intestinal bleeding, ischemia, angina, infarction, and bowel obstruction [12-16]. A previous report suggested that gastrointestinal involvement in NF1 occurs as intestinal ganglioneuromas, GISTs, and carcinoids of the periampullary region of the duodenum that may be associated with pheochromocytoma [3].

Several cases of gastrointestinal adenocarcinomas have been reported in literature [17,18], but the most common gastrointestinal malignancy in NF1 is neuroendocrine tumors in the upper gastrointestinal tract, characteristically in the periampullary region [19-21]. Many are classified as somatostatinomas [22,23], and NF1 has rarely been associated with insulinomas and gastrinomas [24,25]. Our literature review revealed only one reported case of ZES associated with NF1, and hence the present case represents only the second reported case. The multiple small nodular tumors attached to the serosal surface of the small bowel observed during laparotomy in our patient were found to be stromal tumors with a low malignant potential. We assumed that these stromal tumors were an incidental finding that carried no current potential risk of developing into malignant GIST.

Most patients with ZES initially present with abdominal pain, heartburn, diarrhea, and weight loss. These patients are often misdiagnosed as idiopathic peptic ulcer disease, resulting in a delayed true diagnosis that leads to a more advanced stage of the disease.

The diagnosis of ZES is based on previously described criteria [26]. A fasting serum gastrin level of >200 pg/mL should raise the suspicion of the disease, and a level of >1000 pg/mL without the presence of achlorhydria secondary to atrophic gastritis is virtually indicative of ZES. Gastric secretory testing usually demonstrates the presence of a basal acid output of greater than 15 mEq in unoperated patients. The secretin stimulation test is a useful way to confirm the diagnosis of ZES (where the increase is >200 pg/mL), when the fasting serum gastrin level is only modestly elevated and the diagnosis is in doubt [27]. Finally, a positive histologic confirmation of gastrinoma provides definitive evidence of the presence of ZES. In our case, an elevated level of gastrin and characteristic pathologic findings indicated the correct diagnosis of ZES.

More than 80% of the tumors in ZES are located in the so-called gastrinoma triangle [28]. In addition, more than 90% of duodenal gastrinomas occur in the first or second portion of the duodenum, and about 50% of them are solitary [29]. In our case, the tumor was located in the extraduodenal space within the gastrinoma triangle.

Various imaging methods are available for localizing a gastrinoma. In our patient, the tumor was detected by conventional CT and MRI scans as a isolated, well-contained mass without metastasis to the liver or lymph nodes, which enabled curative resection.

## Conclusion

Most patients with NF1 never develop major complications, but the mainstay of care remains anticipatory guidance and surveillance for treatable complications. As in our case, NF1 can be related to neuroendocrine tumors that will progress if not managed early in the course of the disease. As accurate diagnosis of gastrinoma can be difficult, a high level of suspicion and timely evaluation should be applied to NF1 patients manifesting characteristic symptoms attributable to ZES.

## List of abbreviations used

CT: computed tomography

GIST: gastrointestinal stromal tumor

MEN1: multiple endocrine neoplasia type 1

MRI: magnetic resonance imaging

NF1: neurofibromatosis type 1

ZES: Zollinger-Ellison syndrome

## Competing interests

The author(s) declare that they have no competing interests.

## Authors' contributions

CHP, YEJ, HSK, SKC, JSR, and SJK made substantial contributions to the conception and interpretation of clinical data and case-related studies, and clinical decisions. YSK, JCK, and CKC performed the surgery and provided final diagnosis of the disease. All authors read and approved the final manuscript.

## Pre-publication history

The pre-publication history for this paper can be accessed here:
